# Progress in Obstetrics

**Published:** 1900-01-06

**Authors:** 


### PROGRESS IN OBSTETRICS.

**The Operative Treatment of Labour in Cases of Pelvic Deformity.**—Dobbin,[1] from a critical review of the first thousand patients delivered in the obstetrical department of the Johns Hopkins Hospital, draws the following conclusions: (1) In 131 cases of contracted pelves there was necessity for operative delivery in 46; (2) the pelves most frequently requiring operation are the rachitic and irregular forms; (3) pelves in which the degree of contraction is slight and those in which the contraction is very marked are the easiest for treatment, as in both cases the indications are definite, and should give the operator little trouble in deciding upon the treatment to be adopted; (4) on the other hand, the pelves possessing a medium degree of contraction are the most perplexing, and call for the exercise of the greater skill and judgment; when proper appliances are at hand such cases are best treated by tentative application of forceps, and, this failing, immediate Cæsarian section; (5) in general, forceps give a lower fœtal mortality than version, but version done as a primary operation on a movable head in a slightly contracted pelvis is a safer operation for the child than a difficult high forceps operation; except in very exceptional cases symphysiotomy is not to be compared with Cæsarian section, for the former operation, besides causing greater injury to the mother, is always an uncertain procedure. Black,[2] on the other hand, believes that the induction of premature labour combined with symphysiotomy might with advantage take the place of Cæsarian section, the mortality of which is still very great. Davis,[3] of Philadelphia, recommends the following measures in labour with abnormal pelves. He usually employs Simpson's forceps, with Toulet's device of tapes for axis traction, and finds Walcher's position of very decided service, when it becomes necessary to use forceps, before the head has reached the pelvic floor. He limits version to cases of simple flat pelvis, in which contraction is not great, *i.e.*, the internal conjugate must be at least 8 c.m. and upwards, and when the head fails to enter the pelvis. Dr. Walcher[4] strongly recommends the supine position which bears his name for the application of forceps before the head enters the brim, and in extraction after podalic version. When the head has entered the pelvis the extraction ought to be finished either in the obstetric or in the lithotomy position, because, as Professor Lebedoff and Dr. Bartoszewiez[5] observe, the coccypubic diameter is shortest in Walcher's and longest in the lithotomy position.

**The Treatment of Abortion.**—Dr. J. S. Baer[6] considers this subject under three heads, viz.: (1) Threatened abortion; (2) inevitable abortion; (3) incomplete abortion. In the first class of cases the patient should remain in bed in the recumbent position, and keep absolutely quiet; while morphia should be given as required. The fluid extract of Viburnum prunifolium has proved useful in many cases. In the second class he considers the tampon useful if properly applied; it not only diminishes the chance of sepsis, but spares the patient much loss of blood, and by exciting uterine contractions, it often brings the case to a speedy and happy conclusion. The strictest antiseptic precautions should, of course, be adopted, and the tampon removed at the end of 24 hours, when, if abortion has not been completed, instrumental delivery should be resorted to. Coe[7] maintains that while the gauze tamponade and water bags are invaluable for the purpose of softening and dilating the cervix and lower uterine segment, they cannot be depended upon to excite regular uterine contractions, provided the membranes are intact. He believes, however, their use should precede manual dilatation. In the third class, that of incomplete abortion, Baer recommends the use of the curette in preference to the finger, because the latter is more difficult to sterilise, and often cannot be introduced up to the fundus without the use of unwarrantable force. The curetting should be followed by antiseptic irrigation and by packing. Dr. Jenkins[8] calls attention to the extraordinary softness of the uterine tissue after abortion, and records an instructive case in which an extensive rupture of the wall of the uterus, extending into the left parametrium, resulted from dilatation of the cervix for incomplete abortion; and another case where a sound perforated the uterine wall, no undue force being used by the operator. Robinson[9] considers that immediately after abortion, miscarriage, or labour, the sharp curette should not be used. In a septic uterus he says the walls are very friable and extremely easy to perforate. After abortion or labour, perforation by the curette is most frequent and easy at the placental site.

**Occipito-posterior Positions.**—Stricker Coles,[10] writing on posterior rotation of the occiput in vertex presentations, points out that the most common factor in its production is incomplete flexion of the head during its passage through the pelvis; the occipital end being the largest, and passing through the smallest part of the pelvis, seems to meet with greater resistance, while the sinciput becoming the most dependent portion tends to rotate anteriorly. He mentions three cases which were converted into anterior positions by first flexing the head, then grasping it between the thumb and fingers, the patient being placed either on the side towards which the occiput pointed or in the knee-chest position, while by the help of external manipulation the head was rotated anteriorily. Green[11] lays great stress on the importance of posture in the treatment of occipito-posterior positions, and recommends that during the first stage of labour the patient should kneel by the bedside with the body forward over the bed and resting on the arms. This position brings the anterior wall of the uterus lowermost, and in an unengaged occipito-posterior position the back of the fœtus must by force of gravity rotate on its long axis and become dependent. If the head is pocketed in the inlet he advocates at first the knee-chest posture, followed by the kneeling position. If the head has passed the brim, rotation is greatly favoured by the latero-prone posture, the patient lying on the side toward which the occiput should rotate.

[1] Dobbin, New York Med. Jour., Sep. 9, 1899. [2] Black, Glas. Med. Jour., Aug., 1899. [3] Davis, Med. Rec., Sep. 23, 1899. [4] Walcher, Med. Rec., Sep. 23, 1899. [5] Lebedoff and Bartoszewicz, Med. Rec., Sep. 23, 1899. [5] Baer, Med. Rec., July 29, 1899. [7] Coe, Med. Rec., July 8, 1899. [8] Jenkins, Glas. Med. Jour., Aug., 1899. [9] Robinson, Inter. Med. Mag., July, 1899. [10] Stricker Coles, Amer. Jour. of Obstet., Sep., 1899. [11] Green, Inter. Med. Mag., July, 1899.

